# Endobronchial Cartilage Rupture: A Rare Cause of Lobar Collapse

**DOI:** 10.1155/2016/8178129

**Published:** 2016-07-25

**Authors:** Osama Dasa, Nauman Siddiqui, Mohammed Ruzieh, Toseef Javaid

**Affiliations:** University of Toledo, Toledo, OH 43614, USA

## Abstract

Endobronchial cartilage rupture is a rare clinical condition, which can present in patients with severe emphysema with sudden onset shortness of breath. We present a case of a 62-year-old male who presented to our emergency department with sudden onset shortness of breath. Chest X-ray showed lung hyperinflation and a right lung field vague small density. Chest Computed Tomography confirmed the presence of right middle lobe collapse. Bronchoscopy revealed partial right middle lobe atelectasis and an endobronchial cartilage rupture. Endobronchial cartilage rupture is a rare condition that can present as sudden onset shortness of breath due to lobar collapse in patients with emphysema and can be triggered by cough. Bronchoscopic findings include finding a collapsed lung lobe and a visible ruptured endobronchial cartilage. A high index of suspicion, chest imaging, and early bronchoscopy can aid in the diagnosis and help prevent complications.

## 1. Introduction

Spontaneous endobronchial cartilage rupture is a rare clinical condition. To our knowledge there are no reported cases in the medical literature. The only reported cases of bronchial cartilage rupture were clinical scenarios related to traumatic causes including blunt and penetrating chest trauma and double lumen endotracheal intubation [1, 2].

We report a case of spontaneous endobronchial cartilage rupture leading to respiratory failure.

## 2. Case Presentation

62-year-old male with 40 pack-years' history of smoking presented to our emergency department with the chief complaint of sudden onset shortness of breath for five hours. The patient stated that he did have shortness of breath and cough at baseline limiting his activities of daily life but this time dyspnea was with sudden onset and severe. He denied any trauma to chest, fever, chills, chest pain, or recent upper respiratory tract infection.

**Figure 1:**
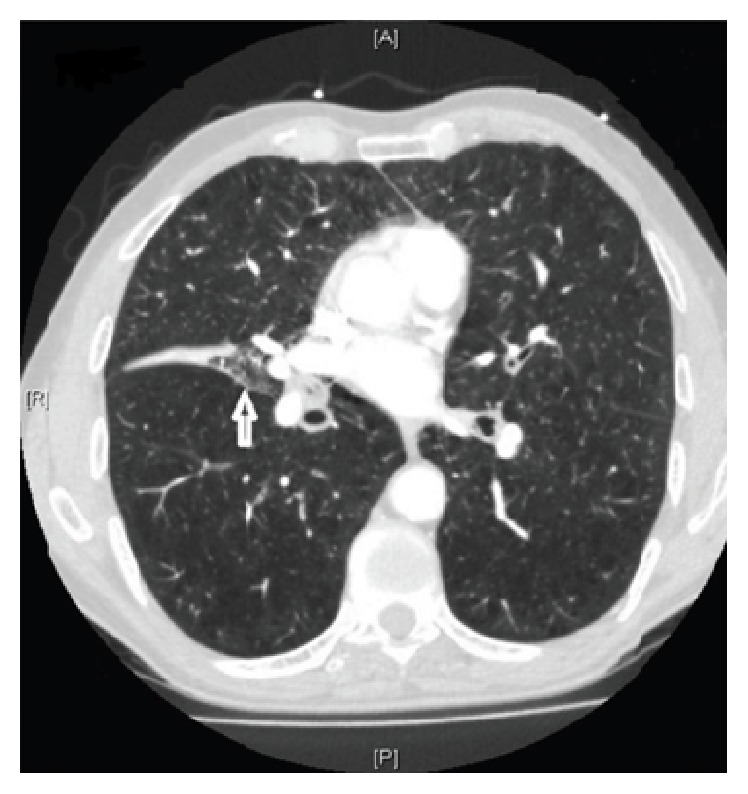
CT-angiogram of the lung showing severe emphysema and near complete collapse of right middle lobe.

**Figure 2:**
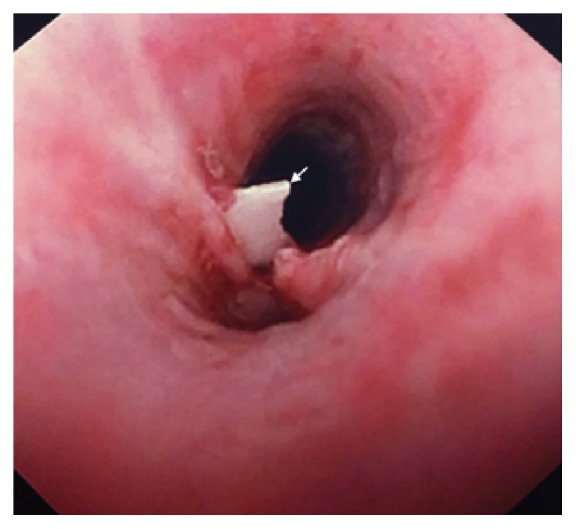
Bronchoscopy revealing rupture of cartilage at subcarina of medial segment of right middle lobe. There was no evidence of endobronchial mass or extrinsic compression.

On physical examination, the patient had tachycardia and tachypnea, with labored breaths and prolonged expiration and rhonchi. Arterial blood gas test showed evidence of hypoxia and hypercapnia. He was started on noninvasive positive pressure ventilation (NIPPV) in emergency department for severe dyspnea. Chest X-ray showed both severe hyperinflation and flattening of diaphragm with vague suggestion of a small density in the right lung field. D-Dimer was 0.74 mcg/ml. Given his sudden onset shortness of breath and clear lung fields and elevated D-Dimer a CT-angiogram was done to rule out pulmonary embolism which showed severe emphysema and near complete collapse of right middle lobe (Figure 1). No extra luminal mass was noticed; however dense material within the bronchus was identified suggestive of mucus plug or intraluminal mass. A bronchoscopy was done to rule out malignancy given patient's extensive smoking history. Bronchoscopy revealed partial right middle lobe atelectasis and rupture of cartilage at subcarina of medial segment of right middle lobe. There was no evidence of endobronchial mass or extrinsic compression (Figure 2). A biopsy was aborted due to possibility of injury to lung parenchymal tissue supported by ruptured cartilage. Patient's shortness of breath improved with conservative management and supportive care.

## 3. Discussion

Tracheobronchial rupture is most frequently described in literature from cardiothoracic surgery and trauma patients (blunt and penetrating) [1–3]. We suggest that in our patient cough may have increased positive expiratory pressure leading to rupture of endobronchial cartilage and subsequent lobar collapse.

The diagnosis of tracheobronchial rupture can be challenging. Symptoms, signs, and investigations can be nonspecific. Presentation depends on the site and size of the air leak and whether it communicates with the pleura. Early surgical primary repair is advocated for most trauma related ruptures to prevent fibrosis and stenosis [3, 4]. In our case, however, a conservative approach was elected, and the patient's symptoms gradually improved, with complete resolution of emphysema and lung collapse. A repeat follow-up bronchoscopy done two months later showed neither evidence of previously noted endobronchial cartilage, nor stenosis or fibrosis.

Spontaneous endobronchial cartilage rupture is a rare clinical condition, which can present in patients with severe emphysema and cough with sudden onset shortness of breath. Bronchoscopic findings include finding a collapsed lung lobe and a visible ruptured endobronchial cartilage. Tracheobronchial injuries are serious and potentially fatal. Presentation is usually nonspecific. A high index of suspicion, chest imaging, and early bronchoscopy can aid in the diagnosis and help prevent complications. In our patient, the endobronchial cartilage did not completely obstruct the lumen raising the possibly that a mucus plug led to complete obstruction of the bronchus.
